# Psycho-socio-economic factors and cardiorenal multimorbidity in middle to older-aged adults: cross-sectional results from the Canadian Longitudinal Study on Aging

**DOI:** 10.1007/s11357-025-01927-9

**Published:** 2025-10-08

**Authors:** Setor K. Kunutsor, Reyhaneh Rikhtehgaran, Anita Soni

**Affiliations:** 1 Section of Cardiology, Department of Internal Medicine, Rady Faculty of Health Sciences, Max Rady College of Medicine, University of ManitobaSt. Boniface Hospital, 409 Tache Avenue, Winnipeg, MB R2H 2A6 Canada; 2 Cardiovascular Real-World Evidence (CARDIO-RWE) Research Group, Section of Cardiology, I.H. Asper Institute, St. Boniface Hospital, 369 Tache Avenue, Winnipeg, MB R2H 2A6 Canada; 3 Department of Physiology and Pathophysiology, Rady Faculty of Health Sciences, University of Manitoba, Manitoba, Canada

**Keywords:** Psycho-socio-economic factor, Cardiorenal multimorbidity, Cross-sectional study, CLSA

## Abstract

Psycho-socio-economic factors (PSEFs) such as income, education, housing, and social support are known to influence health outcomes, yet their relationship with cardiorenal multimorbidity (CRM) remains poorly understood. This study aimed to estimate the prevalence of CRM and examine its associations with PSEFs in a large, nationally representative Canadian sample. We analyzed baseline data from 19,370 participants (mean age: 60 years; 49.8% men) in the Canadian Longitudinal Study on Aging, a prospective cohort of community-dwelling adults aged 45–85 years recruited between 2010 and 2015. CRM was defined as the co-existence of at least one cardiovascular disease and kidney disease. PSEFs assessed included household income, education, homeownership, marital status, employment status, and psychosocial variables. Survey-weighted multivariable logistic regression was used to estimate odds ratios (ORs) and 95% confidence intervals (CIs) for prevalent CRM. The overall prevalence of CRM was 0.83% (160 individuals), equivalent to 3.90 per 1000 individuals (95% CI 2.81–5.41). Prevalence increased with age and was higher among men than women (4.57 vs. 3.35 per 1000) and slightly higher in rural than urban areas (4.47 vs. 3.84 per 1000). Homeownership was associated with significantly lower odds of CRM (OR = 0.56; 95% CI 0.33–0.94). A household income of $50 K–99 K was associated with lower odds of CRM (OR = 0.63; 95% CI 0.39–1.00). No other PSEFs showed clear associations with prevalent CRM. CRM is relatively uncommon but shows variation by age, sex, and geography. Among the PSEFs assessed, homeownership and, to a lesser extent, moderate income were associated with reduced odds of prevalent CRM. These findings highlight the potential role of housing and economic stability in mitigating CRM risk. Longitudinal studies are needed to assess the impact of PSEFs on the development and progression of CRM over time.

**Supplementary Information:**

The online version contains supplementary material available at 10.1007/s11357-025-01927-9.

## Introduction

In Canada, cardiovascular disease (CVD) is the secondleading cause of death, with ischemic heart disease and stroke being the most common contributors[1]. Over 2.4 million Canadian adults live with diagnosed ischemic heart disease, a number expected to rise with the aging population, placing increasing strain on the healthcare system[2]. Chronic kidney disease (CKD) is also a significant concern, affecting at least 4 million Canadians, or roughly 12% of the population[3]. The prevalence is particularly high among Indigenous populations and those of lower socioeconomic status, reflecting systemic healthcare disparities[4]. CKD substantially increases the risk of cardiovascular complications, which are the leading cause of death in this population, and places a heavy burden on the healthcare system[5]. The coexistence of CVD and CKD, referred to as cardiorenal multimorbidity (CRM), is especially problematic due to the synergistic effects of both conditions, leading to increased hospitalizations, mortality, and healthcare costs. CRM disproportionately affects vulnerable groups, including immigrants, and individuals with lower socioeconomic status.

Despite recognition of the individual burden of CVD and CKD, there is a lack of data on the prevalence and sociodemographic variation of CRM in Canada. Understanding how CRM is distributed across age, sex, and geographical location is essential to inform tailored interventions and equitable healthcare delivery.

Traditional risk factors for CVD and CKD are well known, but the role of psycho-socio-economic factors (PSEFs), such as household income, education, employment status, housing stability, social support, and marital status, remains underexplored. These factors influence access to care, lifestyle behaviors, and stress, all of which shape health outcomes[6–10]. Social disadvantage is associated with worse health through multiple pathways, including increased exposure to risk factors and reduced healthcare engagement. Several PSEFs have been shown to independently contribute to the development and progression of CVD and CKD, beyond established biomedical risk factors[11–16]. However, their role in the development of CRM specifically has not been examined. While a few studies have assessed the impact of PSEFs on general multimorbidity[17, 18], there is a gap in understanding their influence on CRM. This study aims to address this knowledge gap by (i) estimating the burden of CRM in the Canadian population, (ii) assessing the associations between multiple PSEFs and prevalent CRM, and (iii) examining whether these associations vary by age, sex, and geographical location.

## Materials and methods

### Study design and population

This study followed the STROBE (Strengthening the Reporting of Observational Studies in Epidemiology) guidelines (Supplementary Material 1) and adhered to the Statistical Analyses and Methods in the Published Literature (SAMPL) guidelines[19]. We conducted a population-based cross-sectional analysis using baseline data from the Canadian Longitudinal Study on Aging (CLSA), collected between 2010 and 2015. The CLSA is a nationally representative cohort of over 50,000 Canadians aged 45 to 85 years, recruited through the Canadian Community Health Survey on Healthy Aging, provincial health registries, and random-digit dialing[20]. Data collection was conducted via two cohorts: the Tracking Cohort (*n* = 21,241), using 60-min computer-assisted telephone interviews, and the Comprehensive Cohort (*n* = 30,097), which included in-person interviews and physical assessments conducted at data collection sites or in participants’ homes. For this study, we used data from the Comprehensive Cohort due to its extensive information on health status, clinical and anthropometric measurements, psychosocial and socioeconomic factors, and blood biomarkers, including lipid profiles. Participants in this cohort were randomly sampled from within a 25–50 km radius of 11 data collection sites across Canada. The CLSA excluded individuals residing in the three territories (Yukon, Northwest Territories, and Nunavut), people living on federal First Nations reserves, full-time Canadian Armed Forces members, and those unable to communicate in English or French. Individuals living in institutions and those with significant cognitive impairment were excluded at baseline. In addition, participants who identified with a gender other than male or female were excluded from our analysis due to limited sample size. Our final analytic sample included participants with complete data on PSEFs, relevant covariates, and CRM status, resulting in a total of 19,370 individuals. Ethics approval for CLSA data collection was granted by 13 research ethics boards across Canada. All participants provided informed consent. Ethics approval for this secondary analysis was obtained from the University of Manitoba Health Research Ethics Board (HS26767).

### Assessment of PSEFs and covariates

Demographic variables considered in the analysis included age, biological sex, and residential location (urbanized vs. rural), determined using participants’ postal codes[21]. Socioeconomic indicators encompassed total household income, educational attainment, homeownership status (classified as own for those who own their dwelling and other for all other arrangements), marital status (categorized as married/common-law or single/previously married—single, widowed, divorced, separated), and employment status (defined as current worker or non-current worker based on the current working status). Missing values for employment status were supplemented using retirement status and reported working hours: individuals who were retired and did not report any work hours were classified as non-current worker, while those who reported any work hours or identified as partly retired were classified as current worker. Psychosocial factors included measures of social support, social participation, social network size, depressive symptoms, loneliness, anxiety disorder, and post-traumatic stress disorder (PTSD). Social support was assessed using the functional social support scale based on the 19-item Medical Outcomes Study (MOS) Social Support Survey, which comprises four subscales: tangible support, emotional/informational support, affectionate support, and positive social interaction[22]. The functional social support scale is scored on a scale from 0 to 100, with higher values reflecting greater levels of perceived support. We divided the score into tertiles, with the first tertile classified as low social support and the second and third tertiles combined as medium–high social support. Social participation is defined as a continuous score consisting of eight variables: family/friends activities out of the household, religious activities, sports or physical activities with others, educational or cultural activities, clubs or fraternal organization activities, association activities, volunteer or charity work, and other recreational activities. For each activity, responses are converted into the monthly number of social activities as follows: “at least one a day,” 20; “at least one a week,” 6; “at least one a month,” 2; “at least one a year,” 1; and “never,” 0. The monthly activity counts are then summed across all categories, with a higher total reflecting greater frequency of participation[23]. Network size is defined as a continuous score based on the sum of the number of living children, living siblings, close friends, and neighbours. A higher score indicates a larger network size[24]. A positive screen for depression symptoms was ascertained using the 10-item version of the Center for Epidemiological Studies Depression Scale (CES-D-10)[25]. Loneliness is measured using the CES-D 10 item on the frequency of feeling lonely, categorized as rarely or never (less than 1 day per week), and felt lonely (1 or more days per week). Behavioural variables included alcohol consumption classified as: never drinker, light or moderate drinker (one to 15 drinks per week for men and one to 10 drinks per week for women), or heavy drinker (15 or more drinks per week for men and 10 or more drinks per week for women)[26] and smoking status, categorized as non-smoker (never smoked a whole cigarette or former occasional smoker) and smoker (current occasional, current daily, or former daily smoker)[26]. Physical activity was assessed using the Physical Activity Scale for the Elderly (PASE), a validated tool that captures engagement in leisure, household, and occupational activities among older adults[27]. The PASE walking frequency variable was recoded into three categories based on the number of days walked per week, in line with broader physical activity research: inactive (no days), insufficiently active (1 to 4 days per week), and sufficiently active (5 to 7 days per week). Blood samples were collected via non-fasting venipuncture at data collection sites and shipped using cryoshippers to Calgary Laboratory Services. Serum levels of total cholesterol, high-density lipoprotein cholesterol (HDL-C), and triglycerides were analyzed by a clinical analyzer (Roche Diagnostics Cobas 8 000 series) at the CLS. Body mass index (BMI) was calculated using measured height and weight: weight in kilograms divided by the square of height in meters (kg/m^2^). Health conditions were identified through self-reported responses to questions on physician-diagnosed chronic illnesses expected to persist for 6 months or more. Diabetes diagnosis indicates self-reported doctor diagnosis of diabetes, borderline diabetes, or high blood sugar. Hypertension was defined based on self-report of a medical diagnosis.

### Ascertainment of outcome

Prevalent CRM was defined as the co-occurrence of any cardiovascular disease (CVD) and kidney disease at baseline (2010–2015). CVDs included angina, myocardial infarction, peripheral vascular disease, stroke, or heart failure. CRM status was categorized as a binary variable (yes/no). Participants were classified as having a cardiorenal condition if they reported ever being diagnosed with each condition by a doctor.

### Statistical analysis

All analyses were weighted to enhance the generalizability of findings to the Canadian population and to account for the complex sampling design of the CLSA. Inflation weights were applied for descriptive statistics, with the exception of frequencies, while analytic weights were used for analytical purposes such as regression analyses, following CLSA guidance[28]. Participant characteristics at baseline were summarized using descriptive statistics. Continuous variables were presented as means with standard deviations (SD) or medians with interquartile ranges (IQR), depending on the distribution’s normality. Categorical variables were summarized using frequencies and percentages. Group comparisons accounted for the complex survey design, using design-adjusted *t*-tests for normally distributed continuous variables, rank-based tests for skewed distributions, and Rao-Scott chi-square tests for categorical variables. Prevalence estimates of CRM per 1000 individuals were calculated overall and stratified by sociodemographic subgroups. To examine associations between PSEFs and CRM, we performed survey-weighted multivariable logistic regression to estimate odds ratios (ORs) and 95% confidence intervals (CIs). Covariates were adjusted for in a stepwise manner across three models: model 1, age and sex; model 2, model 1 plus geographical location, BMI, total cholesterol, HDL-C, smoking, alcohol consumption, hypertension, and diabetes diagnosis; and model 3, model 2 plus physical activity. Covariates were selected based on established associations with cardiometabolic outcomes, previous CLSA-based research[29, 30], and their potential confounding effect on the relationships between PSEFs and CRM[31]. All statistical analyses were conducted using R (version 4.4.2, R Foundation for Statistical Computing, Vienna, Austria).

## Results

Baseline characteristics of the study’s 19,370 participants overall and by CRM status are presented in Table 1. The mean (SD) age of study participants was 60 (10) years, with males constituting 49.8% of the cohort. Participants with prevalent CRM were older, more likely to have a lower household income (< $50 k), be lonely, have depression, and have a diabetes diagnosis and hypertension, and less likely to have post-secondary education, be homeowners, be married, be currently working, have social support, and be a heavy drinker. Participants with prevalent CRM had lower levels of total cholesterol and HDL-C.

**Table 1:** Baseline characteristics of participants overall and by CRM status

**Variable**	**Overall** **(*N* = 19,370)** **Mean (SD) or median (IQR)**	**Not having CRM** **(*N* = 19,210)** **Mean (SD) or median (IQR)**	**Having CRM** **(*N* = 160)** **Mean (SD) or median (IQR)**	***p*-value**
Household income, *n* (%)				< 0.001
< $50 K	4878 (26.2)	4808 (26.1)	70 (49.7)	
$50–99 K	7003 (34.2)	6942 (34.2)	61 (35.4)	
≥ $100 K	7489 (39.6)	7460 (39.7)	29 (14.9)	
Post-secondary education, *n* (%)	16,725 (74.1)	16,595 (74.1)	130 (64.9)	0.03
Homeowner, *n* (%)	16,849 (85.8)	16,728 (85.8)	121 (72.9)	< 0.001
Married/common law, *n* (%)	13,796 (76.4)	13,698 (76.4)	98 (69.9)	0.05
Current worker, *n* (%)	10,234(58.9)	10,195 (59.0)	39 (27.1)	< 0.001
Social support	12,650 (64.6)	12,564 (64.7)	86 (58.6)	0.01
Social participation	15 (10, 22)	15 (10, 22)	16 (10, 26)	0.99
Loneliness, *n* (%)	4662 (25.3)	4607 (25.3)	55 (30.6)	0.02
Depression, *n* (%)	2669 (15.6)	2629 (15.5)	40 (28.8)	0.004
Anxiety, *n* (%)	1554 (8.4)	1538 (8.4)	16 (9.1)	0.21
PTSD, *n* (%)	857 (4.5)	847 (4.5)	10 (8.0)	0.39
Network size	17 (12, 24)	17 (12, 24)	18 (12, 33)	0.52
Males, *n* (%)	9853 (49.8)	9757 (49.7)	96 (57.4)	0.19
Age (years)	60 (10)	60 (10)	70 (10)	< 0.001
Age group				< 0.001
45–54	4984 (38.8)	4975 (39.0)	9 (8.2)	
55–64	6655 (32.6)	6617 (32.7)	38 (24.9)	
65–74	4738 (18.3)	4697 (18.3)	41 (18.2)	
75+	2993 (10.2)	2921 (10.0)	72 (48.7)	
Urbanized area resident, *n* (%)	17,678 (94.2)	17,531 (94.2)	147 (93.9)	0.96
Smoker, *n* (%)	9563 (53.2)	9473 (53.2)	90 (57.9)	0.07
Alcohol, *n* (%)				0.002
Never drinker	4571 (23)	4505 (22.9)	66 (39.6)	
Light/moderate drinker	12,340 (64)	12,257 (64.1)	83 (56.5)	
Heavy drinker	2459 (13)	2448 (13.0)	11 (3.9)	
Physical activity, *n* (%)				0.30
Inactive	2737 (13.7)	2705 (13.7)	32 (21.6)	
Insufficiently active	6475 (33.5)	6418 (33.6)	57 (26.4)	
Sufficiently active	10,158 (52.8)	10,087 (52.8)	71 (52.0)	
Diabetes diagnosis, *n* (%)	3207 (16)	3140 (15.9)	67 (39.9)	< 0.001
Hypertension, *n* (%)	7028 (32.5)	6907 (32.2)	121 (80.1)	< 0.001
BMI (kg/m^2^)	28.16 (5.43)	28.15 (5.43)	28.83 (5.61)	0.16
Total cholesterol (mmol/L)	5.19 (1.06)	5.20 (1.05)	4.44 (1.24)	< 0.001
HDL-C (mmol/L)	1.49 (0.47)	1.49 (0.47)	1.33 (0.40)	< 0.001

Continuous variables are reported as mean (SD) or median (interquartile range) and categorical variables are reported as *n* (%)

Abbreviations: *BMI*, body mass index; *CRM*, cardiorenal multimorbidity; *HDL-C*, high-density lipoprotein cholesterol; *IQR*, interquartile range; *PTSD*, post-traumatic stress disorder; *SD*, standard deviation

Never drinkers were those who reported no alcohol consumption; light/moderate drinkers were those who consumed 1–15 drinks per week for men and 1–10 drinks per week for women; heavy drinkers consumed more than 15 drinks per week for men and more than 10 drinks per week for women. Inactive individuals were those who reported never walking outside; insufficiently active individuals were those walking outside 1–4 days per week; sufficiently active individuals were those walking outside 5–7 days per week. Diabetes diagnosis indicates self-reported doctor diagnosis of diabetes, borderline diabetes, or high blood sugar

Descriptive statistics (except for frequencies) were calculated using inflation weights, while *p*-values were calculated using analytic weights, as per the CLSA protocol, to ensure the results are more representative of the Canadian population

The overall prevalence of CRM was 0.83% (160 individuals), equivalent to an age-, sex-, and location-adjusted prevalence of 3.90 (95% CI: 2.81, 5.41) per 1000 individuals (Table 2). The prevalence of CRM increased with age, with a prevalence of 28.55 (95% CI: 20.87, 38.94) per 1000 individuals in participants aged 75 and over. The prevalence was higher among men than women (4.57 vs. 3.35 per 1000), and slightly higher in rural than urban areas (4.47 vs. 3.84 per 1000).

**Table 2:** Estimated prevalence of CRM per 1000 population, by age group, sex, and location

	Prevalence (95% CI)
Overall	3.90 (2.81, 5.41)
Age group	
45–54	1.30 (0.62, 2.75)
55–64	4.78 (3.27, 6.99)
65–74	9.15 (6.29, 13.30)
75+	28.55 (20.87, 38.94)
Sex	
Men	4.57 (3.28, 6.37)
Women	3.35 (2.18, 5.13)
Location	
Urbanized Area	3.84 (2.77, 5.33)
Rural Area	4.47 (2.17, 9.19)

The associations of PSEFs with the risk of prevalent CRM are presented in Table 3. In age and sex-adjusted analysis, being a homeowner was associated with significantly lower odds of CRM (OR = 0.50; 95% CI 0.30–0.82), which was minimally attenuated to (OR = 0.56; 95% CI 0.33–0.94) on further adjustment for lifestyle factors, lipids, and comorbidities. A household income of $50–99 K was associated with lower odds of CRM (OR = 0.63; 95% CI 0.39–1.00). Initial evidence of lower odds of CRM (OR = 0.54; 95% CI 0.29–1.01) with a household income of ≥ $100 K was attenuated on adjustment for lifestyle factors, lipids, and comorbidities. No other PSEFs showed clear associations with prevalent CRM.

**Table 3:** Associations of PSEFs with the risk of prevalent CRM

PSEFs	Events/total	Model 1		Model 2		Model 3	
		OR (95% CI)	***p***-value	OR (95% CI)	***p***-value	OR (95% CI)	***p***-value
Household Income							
< $50 K	70/4878	ref		ref		ref	
$50–99 K	61/7003	0.61 (0.39–0.96)	.03	0.63 (0.39–1.00)	.05	0.63 (0.39–1.00)	.05
≥ $100 K	29/7489	0.54 (0.29–1.01)	.06	0.65 (0.34–1.22)	.18	0.65 (0.34–1.22)	.18
Education							
No post-secondary education		ref		ref		ref	
Post-secondary education	130/16,725	1.31 (0.84–2.05)	.23	1.47 (0.93–2.32)	.10	1.47 (0.93–2.32)	.10
Homeowner							
No		ref		ref		ref	
Yes	121/16,849	0.50 (0.30–0.82)	.01	0.56 (0.33–0.93)	.03	0.56 (0.33–0.94)	.03
Marital status							
Single/previously married		ref		ref		ref	
Married/common law	98/13,796	1.53 (0.91–2.58)	.11	1.48 (0.87–2.52)	.14	1.49 (0.87–2.54)	.15
Employment status							
Non-current worker		ref		ref		ref	
Current worker	39/10,234	0.80 (0.46–1.40)	.44	0.92 (0.53–1.60)	.77	0.92 (0.53–1.60)	.77
Social support							
Low		ref		ref		ref	
Medium-high	86/12,650	0.81 (0.53–1.24)	.33	0.82 (0.53–1.26)	.36	0.82 (0.53–1.26)	.36
Log of social participation	160/19,370	0.96 (0.78–1.17)	.68	1.00 (0.81–1.23)	.99	1.00 (0.81–1.22)	.98
Loneliness							
Rarely/never (< 1 day/week)		ref		ref		ref	
Felt lonely (≥ 1 day/week)	55/4662	1.25 (0.75–2.08)	.39	1.24 (0.74–2.06)	.42	1.24 (0.74–2.07)	.42
Depression							
No		ref		ref		ref	
Yes	40/2669	1.54 (0.86–2.76)	.15	1.55 (0.87–2.75)	.13	1.55 (0.87–2.75)	.13
Anxiety							
No		ref		ref		ref	
Yes	16/1554	1.43 (0.76–2.70)	.27	1.26 (0.67–2.35)	.47	1.26 (0.67–2.35)	.47
PTSD							
No		ref		ref		ref	
Yes	10/857	1.24 (0.53–2.92)	.61	1.25 (0.54–2.90)	.61	1.25 (0.54–2.88)	.61
Log of network size	160/19,370	1.12 (0.91–1.37)	.27	1.14 (0.92–1.41)	.23	1.14 (0.92–1.41)	.23

*CI*, confidence interval; *OR*, odds ratio; *PSEFs*, psycho-socio-economic factors; *ref*, denotes the reference category used for comparison

Model 1: Age group and sex

Model 2: Model 1 plus location, BMI, total cholesterol, HDL, smoking, alcohol consumption, hypertension, diabetes diagnosis

Model 3: Model 2 plus physical activity

ORs for the log of social participation and the log of network size are per one standard deviation increase

Analytic weights are used in the calculation of ORs and confidence intervals

## Discussion

In this population-based study of over 19,000 adults aged 45–85 years from the CLSA, we found that the prevalence of CRM was relatively low overall (0.83%), corresponding to an adjusted prevalence of 3.90 per 1000 individuals. However, the burden of CRM was disproportionately higher in older adults (notably 28.55 per 1000 among those aged ≥ 75 years), men, and residents of rural areas. Participants with CRM tended to have more adverse psycho-socio-economic profiles, including lower household income, reduced educational attainment, a lack of homeownership, and diminished social support. They also exhibited higher rates of loneliness, depression, and comorbid cardiometabolic conditions such as diabetes diagnosis and hypertension. Among all PSEFs examined, homeownership showed the most consistent inverse association with CRM, even after adjusting for demographic, clinical, and behavioral factors. Household income demonstrated a modest significant association, with lower odds of CRM among those in the $50–99 K income bracket. Other PSEFs did not show clear or statistically significant associations.

Prior research has consistently demonstrated associations between individual PSEFs and the risk of either CVD or CKD. Several systematic reviews and meta-analyses have highlighted the impact of social and economic disadvantage on cardiometabolic outcomes. For example, Valtorta et al. [16] showed in a pooled analysis of 16 datasets that loneliness and social isolation significantly increased the risk of incident coronary heart disease and stroke[16]. Similarly, Zeng et al. conducted a meta-analysis of 43 studies and found that lower income was strongly associated with the prevalence and progression of CKD, while lower education was linked to its prevalence[13]. However, despite this extensive body of work on single-disease outcomes, no prior study, to our knowledge, has specifically explored the associations between PSEFs and CRM as a combined outcome. Our study is among the first to assess the burden of CRM and its relationship with a broad set of PSEFs in a nationally representative Canadian cohort. By estimating CRM prevalence and its relationship with housing, income, and other PSEFs, our findings extend this literature to address an important and understudied area. Notably, our observation that homeownership is independently associated with lower odds of CRM aligns with broader evidence that housing stability confers health benefits, including better chronic disease outcomes[32]. This study thus fills a key knowledge gap and underscores the importance of considering joint disease outcomes in social epidemiology.

The findings from this study have several important clinical and public health implications. The identification of homeownership as a protective factor against CRM underscores the role of housing stability as a key social determinant of health. Interventions that address housing insecurity may contribute not only to individual well-being but also to reducing the burden of chronic disease.

From a public health perspective, although the overall prevalence of CRM is relatively low, it should not be overlooked. The co-occurrence of cardiovascular and kidney diseases represents a clinically serious condition with complex management needs and high healthcare utilization. In the context of an aging population, even a small prevalence can translate into substantial system-wide burden and costs, reinforcing the need for early identification and preventive strategies. Tailored health promotion programs that consider income, education, employment, and social support could help mitigate the development of CRM and associated complications. Furthermore, the absence of strong associations for several other PSEFs suggests a need for more research to understand how these exposures interact over time and across populations. Future longitudinal studies in diverse settings are essential to clarify causal pathways, assess temporal relationships, and guide evidence-based policy interventions aimed at reducing multimorbidity disparities.

This study offers several notable strengths. Most prominently, the use of a large, nationally representative cohort enhances the generalizability of findings to the middle-aged and older Canadian population. The use of the Comprehensive Cohort allowed for the inclusion of detailed health, behavioral, and psychosocial measures, strengthening the depth of exposure assessment. However, some limitations should be acknowledged. First, the cross-sectional design precludes the ability to draw causal inferences between PSEFs and CRM. Second, the absence of information on the timing of disease diagnoses limits our ability to establish temporal sequencing between cardiovascular and kidney disease components. Third, the reliance on self-reported disease status may lead to misclassification, particularly for individuals unaware of their condition, which could underestimate the true prevalence of CRM. This underestimation may, in turn, attenuate observed associations with PSEF exposures. Although prior studies have shown moderate agreement between self-reports and clinical records[33, 34], the potential for both non-differential and differential misclassification remains. Additionally, the CLSA’s exclusion of residents from the territories, federal First Nations reserves, full-time military members, and institutionalized populations may further underestimate disease burden in underserved or high-risk groups. Finally, the relatively small number of CRM events may have limited our statistical power to detect significant associations for less prevalent exposures. Furthermore, we were unable to explore more complex questions such as whether combinations of PSEFs exert synergistic or cumulative effects on CRM risk, whether associations vary across ethnic groups, geographic regions, or age categories, or whether lifestyle factors such as physical activity and diet modify these relationships. Longitudinal analyses using CLSA follow-up data will be needed to assess whether the observed protective effects of homeownership and moderate income persist over time. Such analyses will also provide more power to explore subgroup differences and potential effect modification, which could ultimately inform targeted prevention programs that account for intersecting psycho-socio-economic factors.

## Conclusions

CRM is relatively uncommon but shows variation by age, sex, and geography. Among the PSEFs assessed, homeownership and, to a lesser extent, moderate income were associated with reduced odds of prevalent CRM. These findings highlight the potential role of housing and economic stability in mitigating CRM risk.

## Supplementary Information

Below is the link to the electronic supplementary material.

ESM 1 (DOCX 21.0 KB)

## Data Availability

Data are available from the Canadian Longitudinal Study on Aging (www.clsa-elcv.ca) for researchers who meet the criteria for access to de-identified CLSA data.

## References

[1] Heart Disease in Canada. https://www.canada.ca/en/public-health/services/publications/diseases-conditions/heart-disease-canada.html. Accessed on September 19, 2024.

[2] Report from the Canadian Chronic Disease Surveillance System: Heart Disease in Canada, 2018. https://www.canada.ca/en/public-health/services/publications/diseases-conditions/report-heart-disease-Canada-2018.html. Accessed on September 19, 2024.

[3] Hill NR, Fatoba ST, Oke JL, Hirst JA, O’Callaghan CA, Lasserson DS, et al. Global prevalence of chronic kidney disease - a systematic review and meta-analysis. PLoS ONE. 2016;11:e0158765. 10.1371/journal.pone.0158765. 27383068 10.1371/journal.pone.0158765 PMC4934905

[4] Kelly L, Matsumoto CL, Schreiber Y, Gordon J, Willms H, Olivier C, et al. Prevalence of chronic kidney disease and cardiovascular comorbidities in adults in First Nations communities in northwest Ontario: a retrospective observational study. CMAJ Open. 2019;7:E568–72. 10.9778/cmajo.20190040. 31501170 10.9778/cmajo.20190040 PMC6768774

[5] Manns B, McKenzie SQ, Au F, Gignac PM, Geller LI, Canadians Seeking S, Innovations to Overcome Chronic Kidney Disease N. The financial impact of advanced kidney disease on Canada pension plan and private disability insurance costs. *Can J Kidney Health Dis*. 2017;4:2054358117703986. 10.1177/2054358117703986 10.1177/2054358117703986 PMC5406196 28491340

[6] Wang J, Geng L. Effects of socioeconomic status on physical and psychological health: lifestyle as a mediator. Int J Environ Res Public Health. 2019. 10.3390/ijerph16020281. 30669511 10.3390/ijerph16020281 PMC6352250

[7] Zhang Y, Su D, Chen Y, Tan M, Chen X. Effect of socioeconomic status on the physical and mental health of the elderly: the mediating effect of social participation. BMC Public Health. 2022;22:605. 10.1186/s12889-022-13062-7. 35351078 10.1186/s12889-022-13062-7 PMC8962021

[8] Best JR. Individual socioeconomic status, neighborhood disadvantage, and cognitive aging: a longitudinal analysis of the CLSA. J Am Geriatr Soc. 2024. 10.1111/jgs.19155. 39177423 10.1111/jgs.19155

[9] Kang JW, Oremus M, Dubin J, Tyas SL, Oga-Omenka C. Exploring the differential impacts of social isolation, loneliness, and their combination on the memory of an aging population: a 6-year longitudinal study of the CLSA. Arch Gerontol Geriatr. 2024;125:105483. 10.1016/j.archger.2024.105483. 38788370 10.1016/j.archger.2024.105483

[10] Chamberlain S, Savage RD, Bronskill SE, Griffith LE, Rochon P, Batara J, et al. Retrospective cross-sectional study examining the association between loneliness and unmet healthcare needs among middle-aged and older adults using the Canadian Longitudinal Study of Aging (CLSA). BMJ Open. 2023;13:e068769. 10.1136/bmjopen-2022-068769. 36918248 10.1136/bmjopen-2022-068769 PMC10016309

[11] Isiozor NM, Kunutsor SK, Laukkanen T, Kauhanen J, Laukkanen JA. Marriage dissatisfaction and the risk of sudden cardiac death among men. Am J Cardiol. 2019;123:7–11. 10.1016/j.amjcard.2018.09.033. 30352663 10.1016/j.amjcard.2018.09.033

[12] Wang T, Li Y, Zheng X. Association of socioeconomic status with cardiovascular disease and cardiovascular risk factors: a systematic review and meta-analysis. *Z Gesundh Wiss*. 2023:1–15. 10.1007/s10389-023-01825-4 10.1007/s10389-023-01825-4 PMC9867543 36714072

[13] Zeng X, Liu J, Tao S, Hong HG, Li Y, Fu P. Associations between socioeconomic status and chronic kidney disease: a meta-analysis. J Epidemiol Community Health. 2018;72:270–9. 10.1136/jech-2017-209815. 29437863 10.1136/jech-2017-209815

[14] Albasheer O, Abdelwahab SI, Zaino MR, Altraifi AAA, Hakami N, El-Amin EI, et al. The impact of social isolation and loneliness on cardiovascular disease risk factors: a systematic review, meta-analysis, and bibliometric investigation. Sci Rep. 2024;14:12871. 10.1038/s41598-024-63528-4. 38834606 10.1038/s41598-024-63528-4 PMC11150510

[15] Wang F, Gao Y, Han Z, Yu Y, Long Z, Jiang X, et al. A systematic review and meta-analysis of 90 cohort studies of social isolation, loneliness and mortality. Nat Hum Behav. 2023;7:1307–19. 10.1038/s41562-023-01617-6. 37337095 10.1038/s41562-023-01617-6

[16] Valtorta NK, Kanaan M, Gilbody S, Ronzi S, Hanratty B. Loneliness and social isolation as risk factors for coronary heart disease and stroke: systematic review and meta-analysis of longitudinal observational studies. Heart. 2016;102:1009–16. 10.1136/heartjnl-2015-308790. 27091846 10.1136/heartjnl-2015-308790 PMC4941172

[17] Valenzuela S, Peak KD, Huguet N, Marino M, Schmidt TD, Voss R, et al. Social deprivation and multimorbidity among community-based health center patients in the United States. Prev Chronic Dis. 2024;21:E75. 10.5888/pcd21.240060. 39325637 10.5888/pcd21.240060 PMC11451564

[18] Santhosh R, Kakade SV, Durgawale PM. Determinants of multimorbidity among elderly population in Maharashtra, India: logistic regression analysis. J Educ Health Promot. 2024;13:270. 10.4103/jehp.jehp_1481_23. 39310010 10.4103/jehp.jehp_1481_23 PMC11414868

[19] von Elm E, Altman DG, Egger M, Pocock SJ, Gotzsche PC, Vandenbroucke JP. The strengthening the reporting of observational studies in epidemiology (STROBE) statement: guidelines for reporting observational studies. J Clin Epidemiol. 2008;61:344–9. 10.1016/j.jclinepi.2007.11.008. 18313558 10.1016/j.jclinepi.2007.11.008

[20] Raina P, Wolfson C, Kirkland S, Griffith LE, Balion C, Cossette B, et al. Cohort profile: the Canadian Longitudinal Study on Aging (CLSA). Int J Epidemiol. 2019;48:1752–1753j. 10.1093/ije/dyz173. 31633757 10.1093/ije/dyz173 PMC6929533

[21] Statistics Canada. Population centre and rural area classification 2016. https://www.statcan.gc.ca/eng/subjects/standard/pcrac/2016/introduction. Assessed on Aug 16, 2025. In.

[22] Sherbourne CD, Stewart AL. The MOS social support survey. Soc Sci Med. 1991;32:705–14. 10.1016/0277-9536(91)90150-b. 2035047 10.1016/0277-9536(91)90150-b

[23] Levasseur M, Dubois MF, Genereux M, Naud D, Trottier L, Menec V, et al. Key age-friendly components of municipalities that foster social participation of aging Canadians: results from the Canadian longitudinal study on aging. J Urban Health. 2023;100:1032–42. 10.1007/s11524-023-00762-7. 37594674 10.1007/s11524-023-00762-7 PMC10618123

[24] Deslauriers V, Levasseur M. Typology of social participation and network and health in older adults: results from the Canadian Longitudinal Study on Aging. *J Aging Health*. 2025:8982643241311632. 10.1177/08982643241311632 10.1177/08982643241311632 39787320

[25] Palazuelos-Gonzalez RA, Oude Voshaar RC, Liefbroer AC, Smidt N. Impact of physical activities, sedentarism, and sleep on depression and psychological distress-prospective findings of the Canadian longitudinal study on aging. J Affect Disord. 2025;385:119413. 10.1016/j.jad.2025.119413. 40381855 10.1016/j.jad.2025.119413

[26] Raina P, Ali MU, Joshi D, Gilsing A, Mayhew A, Ma J, et al. The combined effect of behavioural risk factors on disability in aging adults from the Canadian Longitudinal Study on Aging (CLSA). Prev Med. 2021;149:106609. 10.1016/j.ypmed.2021.106609. 33984371 10.1016/j.ypmed.2021.106609

[27] Washburn RA, Smith KW, Jette AM, Janney CA. The physical activity scale for the elderly (PASE): development and evaluation. J Clin Epidemiol. 1993;46:153–62. 10.1016/0895-4356(93)90053-4. 8437031 10.1016/0895-4356(93)90053-4

[28] Canadian Longitudinal Study on Aging (CLSA). 2023 Sampling and computation of response rates and sample weights for the tracking (telephone interview) participants and comprehensive participants (version 2.0). 2023. https://www.clsa-elcv.ca/our-resources/2023-sampling-and-computation-of-response-rates-and-sample-weights-for-the-tracking-telephone-interview-participants-and-comprehensive-participants/. Accessed on August 16, 2025. In.

[29] Fishbook BN, Brinton CD, Siever J, Klassen TD, Sakakibara BM. Cardiometabolic multimorbidity and activity limitation: a cross-sectional study of adults using the Canadian Longitudinal Study on Aging data. Fam Pract. 2022;39:455–63. 10.1093/fampra/cmab129. 34644392 10.1093/fampra/cmab129

[30] Im JHB, Rodrigues R, Anderson KK, Wilk P, Stranges S, Nicholson K. Examining the prevalence and correlates of multimorbidity among community-dwelling older adults: cross-sectional evidence from the Canadian Longitudinal Study on Aging (CLSA) first-follow-up data. Age Ageing. 2022. 10.1093/ageing/afac165. 35930724 10.1093/ageing/afac165

[31] Groenwold RH, Klungel OH, Grobbee DE, Hoes AW. Selection of confounding variables should not be based on observed associations with exposure. Eur J Epidemiol. 2011;26:589–93. 10.1007/s10654-011-9606-1. 21796419 10.1007/s10654-011-9606-1 PMC3168755

[32] Rahman S, Steeb D. Homeownership matters: impact of homeownership on the prevalence of chronic health conditions in the United States. Prev Chronic Dis. 2024;21:E33. 10.5888/pcd21.230324. 38753527 10.5888/pcd21.230324 PMC11155682

[33] Halonen P, Jamsen E, Enroth L, Jylha M. Agreement between self-reported information and health register data on chronic diseases in the oldest old. Clin Epidemiol. 2023;15:785–94. 10.2147/CLEP.S410971. 37396023 10.2147/CLEP.S410971 PMC10312216

[34] Simpson CF, Boyd CM, Carlson MC, Griswold ME, Guralnik JM, Fried LP. Agreement between self-report of disease diagnoses and medical record validation in disabled older women: factors that modify agreement. J Am Geriatr Soc. 2004;52:123–7. 10.1111/j.1532-5415.2004.52021.x. 14687326 10.1111/j.1532-5415.2004.52021.x

